# Practical Points

**Published:** 1896-07-18

**Authors:** 


### PRACTICAL POINTS.

*A Hospital Secretary writes: What is the general rule in small hospitals with reference to the staff finding substitutes when going for a holiday? Do the institutions pay for the locum tenens, or has the official to pay?*

No well-administered hospital fails to make adequate provision for granting sufficient holiday to every member of the staff free of cost to its employés. Indeed, an annual holiday is as necessary and beneficial to the institution as it is to the individual member of the staff, who is entitled to it as a part of his or her emoluments. Some committees make special arrangements that officials supply their own substitute, but it would not be expected unless the arrangements were made beforehand. It is quite an open question, even when a *locum tenens* is supplied at the committee's expense, whether the official or the committee find them.

